# A Real-Life Digital Intervention for Personalized Nutrition in Adults With Overweight or Obesity: Remote Randomized Controlled Trial

**DOI:** 10.2196/73367

**Published:** 2026-01-05

**Authors:** Jelle CBC de Jong, Femke PM Hoevenaars, Lotte GP Peters, Charlotte MM Berendsen, Wilrike J Pasman, Martien PM Caspers, Remon Dulos, Suzan Wopereis

**Affiliations:** 1 Department of Microbiology and Systems Biology Netherlands Organisation for Applied Scientific Research Leiden The Netherlands; 2 Department of Biomedical Signals and Systems University of Twente Enschede The Netherlands

**Keywords:** personalized nutrition, digital, dietary advice, behavior, feedback, digital first, real-world study, remote, do it yourself, DIY

## Abstract

**Background:**

A digital-first strategy is increasingly implemented to reduce participant burden, accelerate recruitment, collect real-world data, and increase the diversity of the study population. However, fully remote studies lack face-to-face interaction, which may affect motivation, particularly in the delivery of personalized nutritional advice. Additionally, self-reported data may vary in terms of standardization and completeness.

**Objective:**

The study’s primary objective is to evaluate the feasibility of conducting a fully remote, fully digital randomized controlled nutritional intervention, including participant experience and the capability to perform do-it-yourself anthropometric measurements at home. Secondary objectives are to determine whether self-collected data could detect changes in body weight and other anthropometric outcomes, and to compare the effectiveness of generic versus personalized nutrition advice, with and without personalized food boxes.

**Methods:**

We conducted a fully online, 3-arm randomized controlled trial including adults with overweight or obesity who were motivated to lose weight. Participants were assigned to a control group that received generic advice (n=43), a personalized intervention group that received personalized advice only (n=40), or a personalized intervention plus group that received personalized advice plus personalized food boxes (n=39). The 6-week intervention was delivered entirely digitally, and all anthropometric measurements, questionnaires, and dietary data were self-collected at home. Feasibility was assessed using adherence metrics, completion of self-measurements, and a user-experience questionnaire. Secondary analyses evaluated weight loss, changes in anthropometry, and exploratory associations, including sex differences.

**Results:**

Feasibility was high—102 out of 122 (83.6%) participants found the self-measured anthropometric assessments easy to perform, and 112 (91.8%) participants reported that completing questionnaires from home was easy. For secondary outcomes, participants receiving personalized, but not generic, nutritional advice significantly lost body weight (–1.0 kg; *P*=.002). Participants receiving personalized food boxes in addition to personalized nutritional advice lost significantly more body weight than the other 2 groups (–2.5 kg; *P*=.001) and also showed a decrease in hip circumference (–2.9 cm; *P*=.01). Personalized advice was not easier or more enjoyable to implement than generic nutritional advice, whereas the addition of personalized food boxes improved the ease of implementing personalized nutritional advice (*P*<.001). All participants, irrespective of the intervention arm, reduced intake of unhealthy food groups, including ready-made meals (113.6 g vs 78.5 g, –30.9%); sauces and gravy (18.8 g vs 10.0 g, –46.8%); sweet snacks (84.8 g vs 64.1 g, –24.4%); savory snacks (50.5 g vs 40.0 g, –20.1%); bread, pasta, rice, and wraps (nutritional quality score of 1.9 vs 1.7, –10.5%); and vegetables (129.0 g vs 118.7 g, –8.0%); and replaced coffee with tea.

**Conclusions:**

This study demonstrates that fully remote, participant-led nutritional intervention studies are feasible, with participants able to independently perform anthropometric measurements and self-report data of sufficient quality to detect meaningful effects. Personalized nutritional advice resulted in greater weight loss than generic advice, and the addition of personalized food boxes further enhanced the beneficial anthropometric effects of the intervention. We conclude that such nutritional intervention studies can be conducted fully online, resulting in measurable anthropometric effects after 6 weeks.

**Trial Registration:**

ClinicalTrials.gov NCT06547983; https://clinicaltrials.gov/ct2/show/NCT06547983

## Introduction

Decentralization of clinical trials is on the rise, meaning that participant inclusion and data collection are performed by the participants themselves, for example, from their homes. This may help attenuate the burden of trial participation, improve participant recruitment, and decrease study dropout [1]. In this way, decentralization helps to avoid insufficient recruitment and underpowered clinical trials [2,3], or complete trial failure [4,5]. Online recruitment and study inclusion may also create opportunities to reach a more diverse population, that is, a better representation of the complete population [6]. In addition, data collected through decentralized trials may represent real-world data, revealing whether interventions work when implemented in real-world settings.

Nutrition studies often require a large sample size, with possibly multiple follow-up measurements and multiple (control) groups. Therefore, decentralization is of interest in this field; however, this may also introduce novel caveats, since protocol adherence and, subsequently, data quality could be jeopardized, possibly leading to false conclusions. A limited number of nutritional studies have indicated that similar results can be produced at home compared with a controlled laboratory setting [7-9]. However, this topic remains understudied, and research is needed to assess participant experience and capability to perform simple measurements from home and fill in questionnaires.

Concomitantly, the paradigm of nutritional intervention studies aiming to improve dietary habits is shifting toward more personalized approaches. Personalized nutrition interventions are tailored for specific individuals based on their individual data, such as phenotype, nutritional habits, and biomarkers [10]. Ideally, personalized nutrition approaches also consider socioeconomic, behavioral, and cultural factors, as well as the food environment [11]. Decentralized studies conducted in real-world settings offer an opportunity to assess external validity and to explore how these contextual factors, such as the local food environment and broader food systems [12], influence intervention effectiveness. Moreover, they provide a unique opportunity to investigate behavioral patterns within the food environment and the food systems framework.

Studies have shown that such personalized nutritional advice is more effective than a generic one-size-fits-all approach in improving dietary habits [10,13] and in promoting weight loss in individuals with obesity [14,15]. However, more research is needed in this field to understand the contexts in which personalized nutrition is most effective [14]. Some studies have suggested that personalized nutrition is more effective than generic advice due to its face-to-face aspect and its perception as personally relevant [16,17]. Consequently, it can be questioned whether personalized nutrition interventions are as effective when delivered through the internet, possibly failing to provide recipients with the same level of motivation. Therefore, we chose to test the effectiveness of personalized versus generic nutritional interventions in this real-life, fully remote study.

In summary, the primary objective of this study was to evaluate the feasibility of conducting a fully remote, fully digital nutritional intervention, including participants’ experience and their ability to perform do-it-yourself anthropometric measurements from home. In addition, feasibility was evaluated by examining the reliability of self-reported anthropometric data through analyses of correlations among changes in body weight, waist circumference, and hip circumference. Feasibility was defined as the capability of participants to independently perform all study procedures, including self-measured anthropometry and digital questionnaire completion, in a fully remote setting. Secondary objectives were to assess whether significant changes in body weight and other anthropometric outcomes could be detected using self-collected data, and to compare the effectiveness of generic versus personalized nutritional advice delivered digitally. In addition, we explored whether the provision of personalized food boxes enhanced adherence and led to greater weight loss, and we conducted exploratory analyses of sex differences.

## Methods

### Ethics Statement

This study was reviewed and approved by the independent Internal Ethical Review Board of The Netherlands Organization for Applied Scientific Research (TNO), registration number #2023-083, in September 2023. The study was registered at ClinicalTrials.gov (NCT06547983). All participants provided written informed consent before inclusion in the study. The study was conducted in accordance with the Declaration of Helsinki. All analyses presented in this paper were conducted in accordance with the primary approved study protocol. All data were anonymized. Each participant received a Christmas food box (valued at €90 [US $106]) as compensation for their participation in the trial. Additionally, depending on group allocation, some participants received free daily meals during the trial.

### Study Population

Participants were 25-60 years of age and had a BMI between 25 and 40 kg/m^2^. Participants were included only if they were motivated to lose weight; had the skills to complete digital questionnaires; and had a computer and a smartphone, a weighing scale, and a measuring tape to perform the anthropometric measurements, as confirmed in the screening questionnaire (Multimedia Appendix 1). Participants were excluded if they had a food allergy, followed specific diets (eg, a keto or vegan diet), suffered from a chronic disease that influences food intake (eg, inflammatory bowel disease), or participated in another intervention study. Females in menopause transition, as defined through the screening questionnaire, were excluded as well. A formal power calculation was not feasible because reliable estimates of effect size and variance for this specific intervention and outcomes were not available at the time of study design. Instead, the sample size was based on the results of previous studies [13,18,19], in which 37, 59, or 82 participants were included and exposed to personalized nutritional advice for 16, 9, or 10 weeks, respectively. Consequently, a sample size of 150 at the start of the intervention was considered acceptable for this study. This number was higher than those in the cited previous studies due to the shorter intervention period (6 weeks), which was expected to require a larger sample size. To compensate for an anticipated 10%-20% dropout rate during the intervention, a total sample size of 180 participants was selected.

Participant recruitment was organized via advertisements on social media, as well as through the Dutch participant database of Norstat [20], focusing on zip codes to which food boxes could be delivered (Uitgekookt Meal Service). Only individuals who indicated that their motivation to participate in the study was to lose weight were selected (participants could select 1 of 4 predefined reasons). These participants were randomly allocated to 1 of 3 intervention groups, while taking into account the covariates biological sex, age, and BMI. Allocation was performed by calculating the Euclidean distance for the variable sex and the Kullback-Leibler divergence for the variables age and BMI for each permutation separately. The permutation with the best overall score (ie, the most balanced distribution among the groups) was selected. This allocation process was automated and blinded to both participants and investigators involved in data collection and analysis.

### Study Design

**Figure 1:**
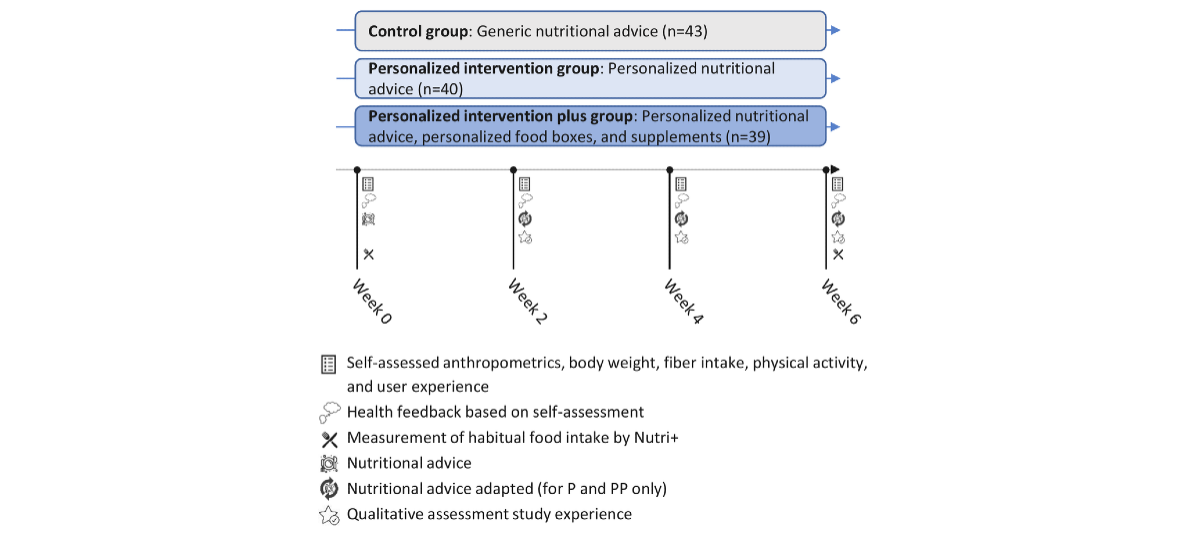
Overview of the study design. Self-measured anthropometric data, including body weight, and assessments of fiber intake and physical activity (via questionnaires) were collected through the online How Am I app at weeks 0, 2, 4, and 6. All intervention groups received feedback on their self-assessments at weeks 0, 2, 4, and 6. Nutritional advice provided to intervention groups P and PP was updated based on participants’ self-assessments. Qualitative assessments of study experience were collected at weeks 2, 4, and 6. Habitual food intake was assessed at the beginning and end of the trial using the Nutri+ module. P-group: personalized intervention group; PP-group: personalized plus intervention group.

The primary objective of this study was to evaluate the feasibility of conducting a fully remote, fully digital nutritional intervention trial, including participants’ ability to perform do-it-yourself anthropometric measurements, adherence to the protocol, and user experience. To further evaluate feasibility, the reliability of self-reported anthropometric data was examined by analyzing correlations among changes in body weight, waist circumference, and hip circumference. Secondary objectives were to assess whether self-collected data could detect significant changes in body weight and other anthropometric measures, and to compare the effectiveness of generic versus personalized nutritional advice, with and without personalized food boxes. Exploratory analyses included examining sex differences to assess the reliability of the self-assessed anthropometric data. The study was a randomized controlled trial with 1 control intervention arm and 2 personalized intervention arms (Figure 1). The study duration was 6 weeks. Participants allocated to the control group received generic nutritional advice (see example in Multimedia Appendix 2), which was based on the Dutch national guidelines for healthy nutrition, assembled by the Dutch Health Council (Gezondheidsraad), an independent scientific advisory body for the Dutch government.

### Study Intervention

Participants allocated to the personalized intervention group (P-group) received personalized advice instead of generic advice. The personalized advice entailed information on the specific number of calories to consume on a daily basis to meet their personal goal of weight loss. Furthermore, the proportion of macronutrients the participant should consume to reach their weight loss goal was also made explicit in the nutritional advice (see example in Multimedia Appendix 2). The prescribed caloric intake provided a daily energy deficit of 400 kcal below the daily energy requirement, as calculated using the Ten Haaf Formula [21] at the start of the trial, based on personal information including age, sex, and physical activity level (PAL). In addition, instructions were provided for the implementation of the Eetmeter application (Voedingscentrum [22]), a freely available digital application that participants were advised to use to check whether their daily meals matched their personalized nutritional advice according to the Dutch dietary guidelines.

Participants allocated to the personalized plus intervention group (PP-group) received the same personalized advice as the P-group; however, they also received personalized food boxes containing meals that were composed to match the personalized nutritional advice in terms of calories and macronutrients. These food boxes contained 3 main meals (breakfast, lunch, and dinner) and 3 snacks to be consumed between the main meals (Uitgekookt Meal Service, IJsselmuiden). The food boxes provided a daily energy deficit of 200-600 kcal below the daily energy requirement. The food boxes were provided on 5 out of 7 days per week. On the remaining 2 days, participants were instructed to use the online Eetmeter application to select and follow their diet based on the provided personalized advice.

Participants allocated to groups receiving personalized interventions received updated nutritional advice based on the outcomes of the self-measurements in terms of calories and macronutrients, whereas the control group received the same generic advice at weeks 2, 4, and 6.

### Remote Data Collection

The study was fully digital, without any face-to-face contact between participants and researchers. To this end, participants were required to install the online research application, the How Am I app (TNO), through which all instructions, feedback, nutritional advice, questionnaires, and reminders were provided, and which served as the data collection platform [23]. At weeks 0, 2, 4, and 6, participants submitted data via the How Am I app on feasibility-related user experience items, self-measured anthropometric measurements, body weight, and brief questions related to fiber intake and physical activity (Multimedia Appendix 1). PAL and fiber intake were calculated according to validated methodologies, as described by Healey et al [24] and in the Food and Agriculture Organization (FAO)/World Health Organization (WHO)/United Nations University (UNU) Expert Consultation Report [25]. At weeks 2, 4, and 6, all participants received a reminder via the TNO How Am I app to complete all anthropometric measurements and questions and were provided with feedback on their measurements (see Multimedia Appendix 2). Researchers monitored data entry, issuing a reminder to participants who did not complete their questionnaires within 48 hours and instructing them to respond within an additional 24-hour period. Participants who remained noncompliant after this interval were excluded from the study.

### Feasibility Outcomes

The primary outcome of this study was the feasibility of conducting a fully remote, fully digital nutritional intervention. Feasibility was assessed at weeks 2, 4, and 6 through participants’ reported user experience, including ease of performing anthropometric measurements, clarity of instructions, and perceived burden, measured using multiple-choice questions, Likert scales, and visual analog scales (see Multimedia Appendix 1). Feasibility was further evaluated by examining the reliability of self-reported anthropometric data, based on correlations among changes in body weight, waist circumference, and hip circumference.

### Self-Measured Anthropometrics and Body Weight

Secondary outcomes related to anthropometrics and body weight were also evaluated. At baseline and at weeks 2, 4, and 6, all participants were requested to self-measure body weight (kg). Using a measuring tape, participants were asked to measure height (cm), waist circumference (cm), and hip circumference (cm) according to standard operating procedures [26]. Detailed instructions on performing the anthropometric measurements were provided via the TNO How Am I app through videos containing step-by-step guidance. Body weight and height were used to calculate BMI (kg/m^2^).

### Measurement of Habitual Dietary Intake

At the start and end of the trial, all participants completed the Nutri+ module (TNO), a web-based questionnaire consisting of 89 questions that measures the dietary intake of 21 food groups for each participant and thereby estimates habitual food intake. Consequently, the average food intake for certain food groups (eg, fruit, meat, dairy) or the average frequency of consumption per week (eg, bread, potatoes, cooking fats) was calculated.

### Statistical Analysis

All analyses were conducted using GraphPad Prism 10 (GraphPad Software) or IBM SPSS Statistics 29. Outlier analysis was performed, and data points greater than 3× the IQR were excluded. As the study aimed to evaluate intervention efficacy based on complete longitudinal data, analyses were performed per protocol. Participants with incomplete datasets (eg, due to dropping out) were not included in the analysis, as missing repeated-measures data would have violated model assumptions and precluded valid within-participant comparisons over time. Normality was confirmed using the D’Agostino and Pearson test. All data are reported as means (SD). Two-tailed *P* values were used, and *P* values <.05 were considered statistically significant.

User experience data collected via Likert scales, multiple-choice questions, and visual analog scales were analyzed descriptively. Group differences in categorical or ordinal feasibility responses were evaluated using Pearson chi-square tests, with Bonferroni-adjusted post hoc tests applied when appropriate. The internal validity of self-reported anthropometric measurements was examined through Pearson correlations among changes in body weight, waist circumference, and hip circumference.

Differences between groups at baseline were tested using one-way ANOVA with Tukey post hoc tests. Anthropometric outcomes collected across multiple time points (weeks 0, 2, 4, and 6) were analyzed using repeated-measures ANOVA, with “time” as a within-participant factor and “group” as a between-participant factor. The Tukey post hoc tests were performed when main effects or interactions were detected. Categorical variables were compared using Pearson chi-square tests with Bonferroni corrections. Sex-stratified analyses were also performed to explore potential sex differences in anthropometric changes by testing for significant interaction effects with sex. Correlations between variables were assessed using the Pearson correlation coefficient, and statistical significance was defined as *P*<.05.

## Results

### Baseline Study Population Characteristics

**Figure 2:**
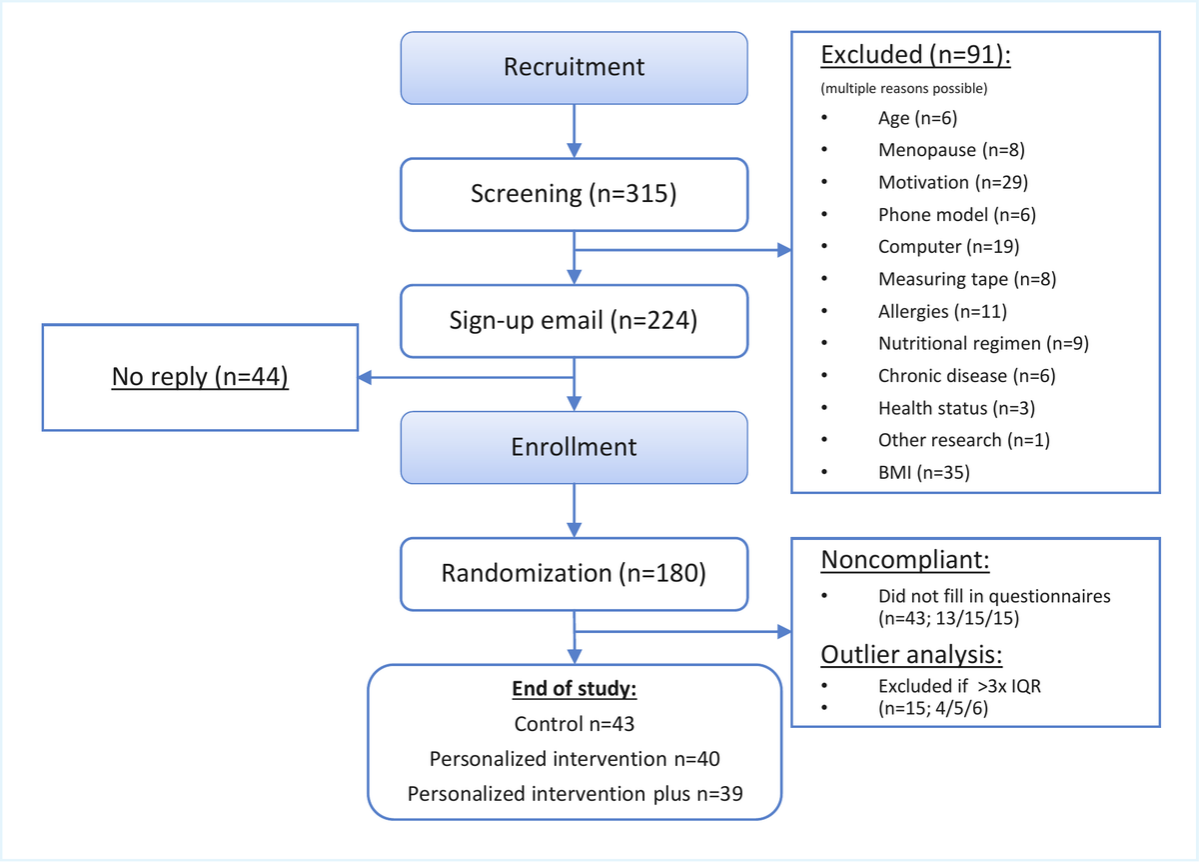
A flowchart of the recruitment and selection process, randomization, and group allocation of included participants.

A total of 315 participants were screened, and of these, 91 were excluded from participation for various reasons (Figure 2). The remaining 224 participants were invited to participate in the study, of whom 180 responded positively and were included in the study and randomly allocated to 1 of the 3 groups. Of the 180 recruited participants, 43 did not respond to the questionnaires and were consequently excluded from the study (Figure 2). Data from the remaining 137 participants were included in the analysis. An additional 15 participants were excluded based on outlier analysis, resulting in a final sample size of 122 participants. Results are reported in accordance with CONSORT (Consolidated Standards of Reporting Trials)-EHEALTH recommendations (Multimedia Appendix 3).

No statistically significant differences in any baseline characteristics were detected among the 3 intervention groups (Table 1). Habitual food intake was also measured for each food group before the intervention (Table 2). No statistically significant differences in habitual food intake were detected among the 3 intervention groups for any food group, except for sauces and gravy. Similarly, no statistically significant differences were found in baseline PAL among the 3 groups (Table 3; *P*=.15).

**Table 1:** Baseline characteristics of the study population.

Characteristics	Control (n=43)	Personalized intervention (n=40)	Personalized plus intervention (n=39)	*P* value
Age (years), mean (SD)	39.9 (6.1)	41.0 (6.0)	40.9 (5.9)	.60
Female sex, n (%)	27 (63)	27 (68)	19 (49)	.18
Weight (kg), mean (SD)	89.9 (12.0)	89.5 (13.3)	92.4 (14.1)	.65
Height (m), mean (SD)	1.75 (0.09)	1.75 (0.11)	1.75 (0.10)	.54
BMI (kg/m^2^), mean (SD)	30.4 (4.1)	30.2 (4.5)	31.2 (4.8)	.81
Waist circumference (cm), mean (SD)	99.0 (10.1)	101.3 (10.0)	103.0 (12.4)	.26
Hip circumference (cm), mean (SD)	108.3 (10.1)	108.3 (7.5)	110.8 (8.9)	.36
Self-perceived health (out of 5 points, 5 being the highest score), mean (SD)	3.0 (0.6)	3.2 (0.7)	3.0 (0.8)	.48
Physical activity level, mean (SD)	1.40 (0.2)	1.44 (0.1)	1.40 (0.1)	.15
Fiber intake (g), mean (SD)	11.3 (5.2)	12.4 (8.4)	12.5 (10.6)	.99

**Table 2:** Baseline habitual food intake (based on the Nutri+ module) per food group of the study population.^a^

Food group	Control (n=43), mean (SD)	Personalized intervention (n=40), mean (SD)	Personalized plus intervention (n=39), mean (SD)	*P* value
Fruit (g/day)	141.6 (110.3)	136.7 (130.6)	113.3 (95.4)	.53
Vegetables (g/day)	122.3 (47.6)	132.6 (56.7)	125.2 (63.4)	.69
Legumes (g/week)	172.3 (284.0)	148.1 (202.1)	151.2 (161.6)	.91
Bread (nutritional quality score)	1.8 (0.6)	1.8 (0.7)	2.1 (0.7)	.09
Pasta, rice, and wraps (nutritional quality score)	1.8 (0.9)	1.9 (0.9)	2.1 (0.7)	.23
Potatoes (nutritional quality score)	2.4 (1.5)	2.7 (1.7)	2.2 (1.3)	.36
Meat (g/week)	2367.9 (1737.8)	2422.8 (1336.4)	2584.9 (1650.3)	.79
Fish (g/week)	348.8 (701.5)	223.1 (242.1)	218.9 (232.9)	.40
Eggs (g/week)	209.5 (305.1)	263.8 (210.6)	233.8 (163.3)	.60
Dairy (g/day)	314.6 (573.5)	308.3 (301.5)	197.3 (193.3)	.39
Nuts (g/day)	15.1 (22.7)	12.9 (16.4)	8.3 (10.3)	.24
Spreadable fats (nutritional quality score)	1.1 (0.9)	1.2 (1.0)	1.1 (0.8)	.94
Cooking fats (nutritional quality score)	1.5 (0.6)	1.3 (0.7)	1.4 (0.8)	.47
Sweet snacks (g/week)	77.6 (83.3)	83.4 (83.9)	94.3 (68.6)	.64
Savory snacks (g/week)	55.6 (74.0)	41.8 (37.4)	54.3 (49.4)	.39
Ready-made meals (g/week)	127.3 (218.5)	112.9 (85.9)	98.8 (97.2)	.73
Sauces and gravy (g/week)	15.0 (20.6)^b^	24.5 (22.8)^b^	17.0 (17.1)^b^	.02
Alcohol (units drank/day)	0.7 (1.1)	0.7 (1.2)	0.5 (0.8)	.82
Soft drinks and fruit juice (g/day)	209.4 (374.2)	176.1 (272.0)	278.3 (489.4)	.49
Tea (g/day)	453.1 (448.1)	360.7 (390.7)	400.2 (399.0)	.63
Coffee (g/day)	424.7 (476.0)	338.8 (378.1)	403.5 (451.5)	.67

^a^Data represent the average habitual food intake. Nutritional quality scores reflect factors such as whether whole wheat versus multigrain products were consumed or whether olive oil versus butter is used for frying. A lower score represents a healthier food choice. Extensive descriptions of these nutritional quality scores are attached as Multimedia Appendix 4.

^b^Control versus personalized intervention, *P*=.02; control versus personalized plus intervention, *P*=.54; personalized intervention versus personalized plus intervention, *P*=.23.

**Table 3:** Physical activity levels during the study period.

Physical activity level	Week 0	Week 2	Week 4	Week 6
Control group, mean (SD)	1.40 (0.2)	1.40 (0.2)	1.42 (0.2)	1.41 (0.2)
Personalized intervention, mean (SD)	1.44 (0.1)	1.45 (0.1)	1.44 (0.1)	1.45 (0.1)
Personalized plus intervention, mean (SD)	1.40 (0.1)	1.41 (0.1)	1.40 (0.1)	1.40 (0.1)

### Participant-Reported Usability and Reliability of Measurements

**Figure 3:**
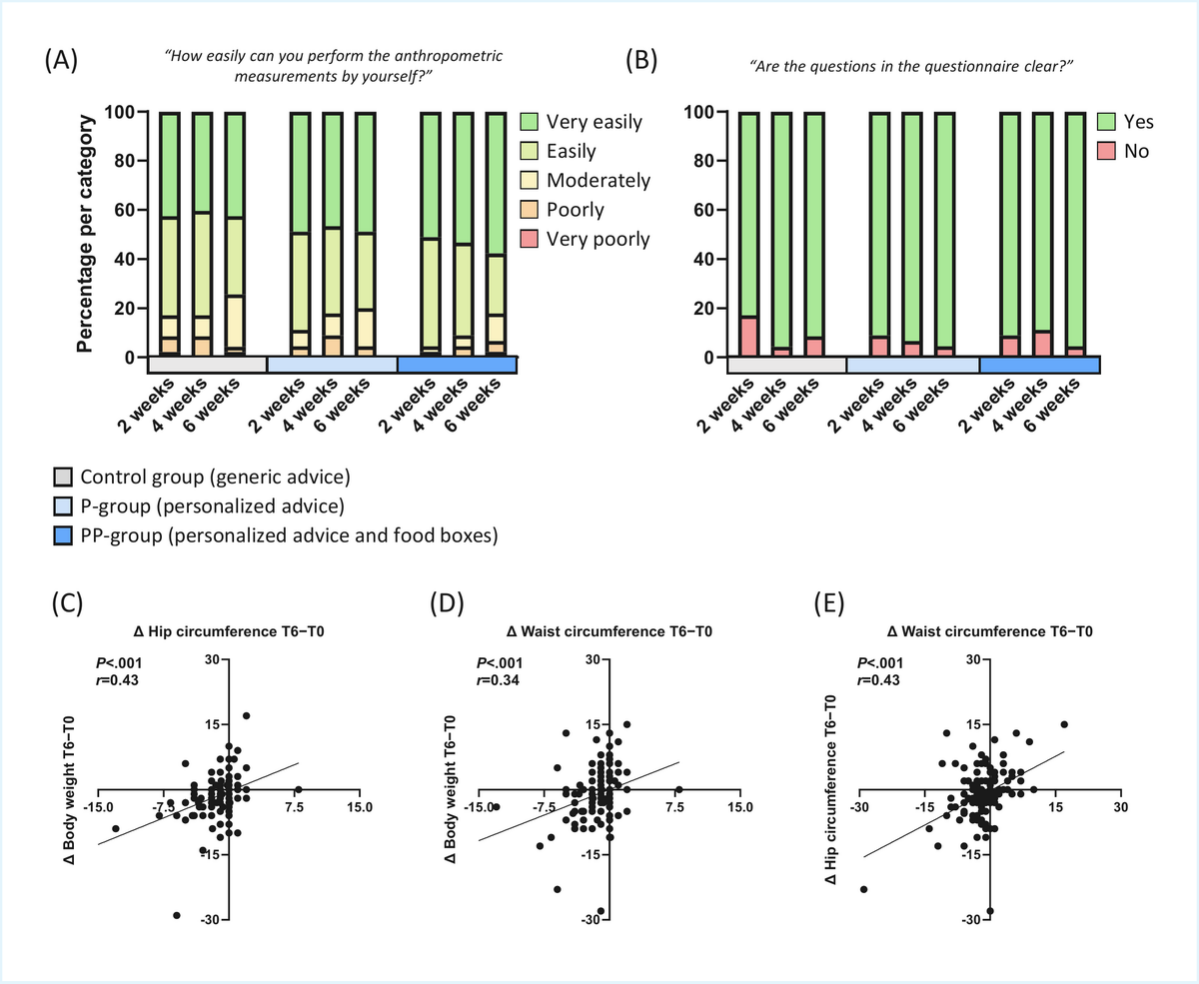
Ease and ability to perform anthropometric measurements at home and to complete questionnaires. (A) Ease of independently performing anthropometric measurements. (B) Clarity of the questionnaires. (C) Correlation between Δ body weight and Δ waist circumference. (D) Correlation between Δ body weight and Δ hip circumference. (E) Correlation between Δ waist and Δ hip circumference. In all correlation analyses, Δ values were calculated as the difference between measurements at the beginning and end of the 6-week trial. P-group: personalized intervention group; PP-group: personalized plus intervention group.

The ease of collecting anthropometric data by participants themselves and completing the questionnaires digitally via the How Am I app was reported (Multimedia Appendix 1). The majority of participants indicated that they could perform the anthropometric measurements very easily or easily (102/122, 83.6%, participants across all groups; Figure 3A), with no differences among groups. In addition, most participants reported that they could understand the questionnaires (112/122, 91.8%, participants across all groups; Figure 3B). The quality of anthropometric data was cross-validated, as significant correlations were detected between changes in body weight, waist circumference, and hip circumference (week 6 to week 0; all *P*<.001, *r*>0.34; Figure 3C-3E).

### Trial Experience and Self-Reported Dietary Changes

**Figure 4:**
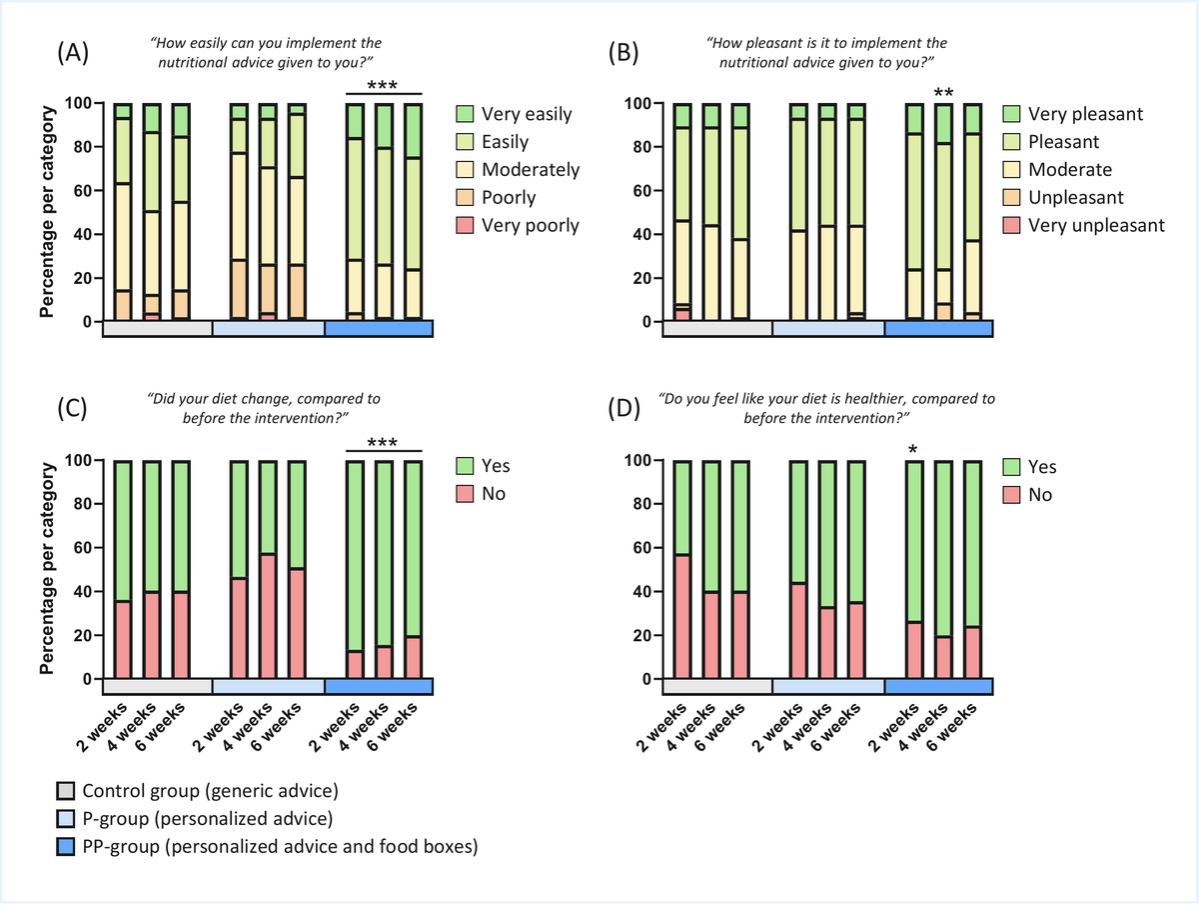
Participants' experience of participating in the fully digital trial. (A) Ease of implementing the nutritional advice during the trial. (B) Enjoyment of implementing the nutritional advice during the trial. (C) Self-reported changes in participants' diets during the trial. (D) Self-reported improvements in dietary healthfulness during the trial. *P<.05, **P<.01, ***P<.001. P-group: personalized intervention group; PP-group: personalized plus intervention group.

No significant differences were detected in the experienced ease of implementing generic versus personalized nutritional advice during the trial (*P*=.43; Figure 4A). However, participants in the PP-group, who received personalized food boxes in addition to the personalized nutritional advice, reported that they could implement the nutritional advice more easily (*P*<.001; Figure 4A). At week 4 only, participants in the PP-group reported experiencing a greater amount of pleasure while implementing their nutritional advice (*P*<.001 at week 4; Figure 4B), whereas no differences were observed between the groups receiving generic versus personalized nutritional advice. No differences in self-reported changes in diet were detected between the control group and the P-group (Figure 4C), while the PP-group consistently reported significant changes in their diet during the trial (*P*<.001; Figure 4C). Similarly, the PP-group reported significantly more often that they assessed their diet as being healthier at week 2 (*P*=.01; Figure 4D).

### Changes in Body Weight, BMI, and Anthropometric Measures

**Figure 5:**
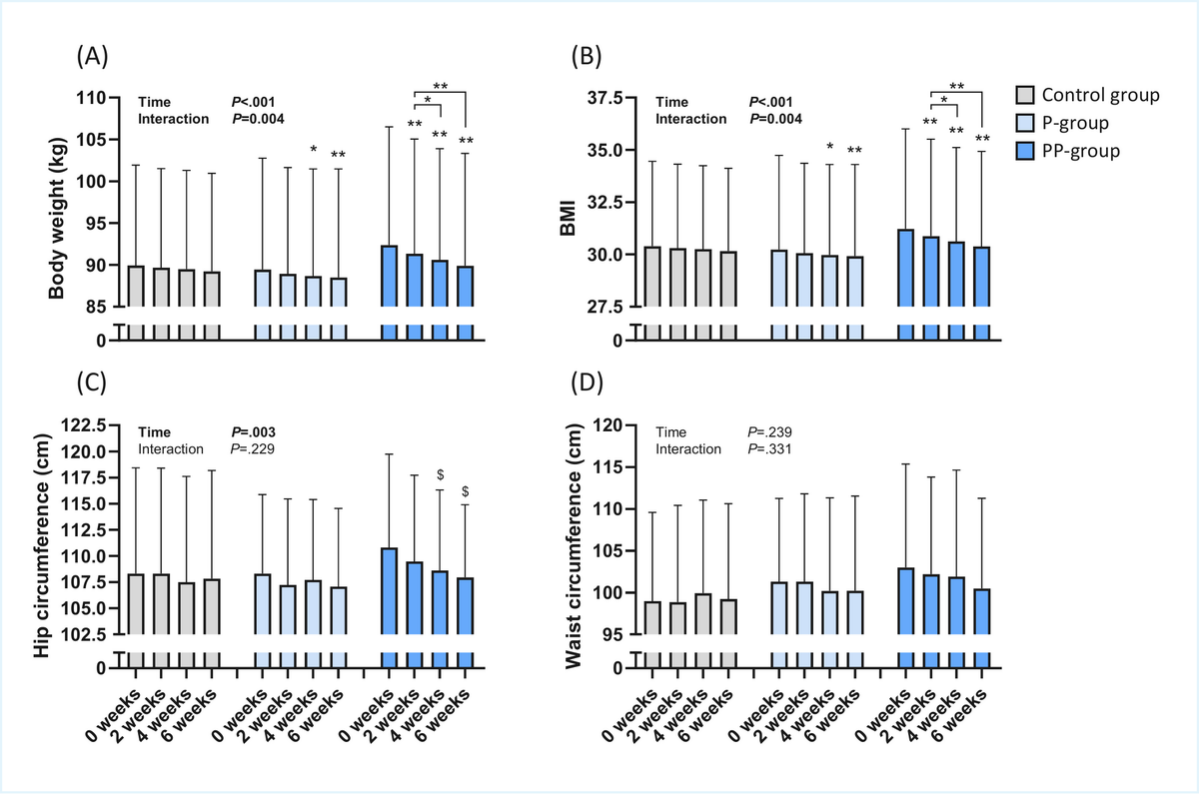
Effects of the different interventions on anthropometric measurements. (A) Body weight, (B) BMI, (C) hip circumference, and (D) waist circumference during the trial. Data are expressed as means (SDs). *P<.05 and **P<.01. The $ symbols indicate significant differences attributable to a statistically significant time effect in the absence of an interaction effect. P-group: personalized intervention group; PP-group: personalized plus intervention group.

After 6 weeks, the average body weight of the control group did not change compared with the start of the trial (*P*=.06; Figure 5A). In the P-group, body weight was significantly lower at week 4 (–0.8 kg; *P*=.04) and week 6 (–1 kg; *P*=.002) compared with baseline (Figure 5A). In the PP-group, body weight was significantly lower at week 2 (–0.9 kg; *P*=.004), week 4 (–1.6 kg; *P*=.002), and week 6 (–2.3 kg; *P*=.001) compared with baseline (Figure 5A). With respect to BMI, similar differences between the groups were observed (Figure 5B). BMI decreased significantly more in the PP-group (–0.8 kg/m^2^, *P*=.001) compared with the P-group (–0.3 kg/m^2^; *P*=.002) and the control group (–0.2 kg/m^2^; *P*=.06) at week 6 (Figure 5B).

Hip circumference decreased significantly over time (*P*=.01), but no significant interaction between time and group was detected (*P*=.22; Figure 5C). Post hoc analysis revealed that the time effect was mainly driven by a significant loss of hip circumference in the PP-group, which decreased at week 4 (–2.2 cm; *P*=.04) and week 6 (–2.9 cm; *P*=.01) compared with baseline (Figure 5C). No significant effects were observed on waist circumference (*P*_interaction_ =.33, *P*_time_=.24; Figure 5D).

### Changes in Habitual Food Intake and Physical Activity

Changes in intake of the food groups vegetables, bread, pasta/rice/wraps, sweet snacks, savory snacks, ready-made meals, sauces and gravy, tea, and coffee were observed (Table 4). The intake of all these food groups was lower after 6 weeks compared with baseline (vegetables, –9.0 g; bread, –0.2 times eaten per week; pasta/rice/wraps, –0.2 times eaten per week; sweet snacks, –21.1 g; savory snacks, –10.7 g; ready-made meals, –60.3 g; sauces and gravy, –8.7 g; coffee, –62.1 g), except tea, which significantly increased (+64.5 g; Table 4). No changes were detected for the food groups fruit, legumes, potatoes, meat, fish, eggs, dairy, nuts, spreadable and cooking fats, alcohol, or soft drinks and fruit juice. Only time effects were significant (see Table 4), without interaction effects, implying that changes in food intake occurred across all intervention groups, but no group-specific effects were observed. Only the food group legumes nearly reached statistical significance for both personalized interventions compared with the control group (*P*=.051), with increased intake postintervention for both the P- and PP-groups, whereas the control group, on average, showed decreased intake postintervention. Fiber intake was measured every 2 weeks, but no changes were detected (Multimedia Appendix 5). In addition, PALs did not change during the study (Table 3).

**Table 4:** Habitual food intake per food group before and after the intervention.a

Food group	Control (preintervention), mean (SD)	Control (postintervention), mean (SD)	Personalized intervention (preintervention), mean (SD)	Personalized intervention (postintervention), mean (SD)	Personalized plus intervention (preintervention), mean (SD)	Personalized plus intervention (postintervention), mean (SD)	Time effect (*P* value)	Interaction effect (*P* value)
Fruit (g/day)	141.6 (110.3)	142.5 (101.4)	136.7 (130.6)	128.8 (136.5)	113.3 (95.4)	105.5 (82.4)	.64	.92
Vegetables (g/day)	122.3 (47.6)	113.4 (44.0)	132.6 (56.7)	116 (52.0)	125.2 (63.4)	123.6 (57.6)	.03^b^	.35
Legumes (g/week)	172.3 (284.0)	121.7 (166.8)	148.1 (202.1)	172.0 (266.3)	151.2 (161.6)	183.6 (225.3)	.90	.051
Bread (nutritional quality score)	1.8 (0.6)	1.6 (0.7)	1.8 (0.7)	1.7 (0.7)	2.1 (0.7)	1.9 (0.6)	.02^b^	.37
Pasta, rice, and wraps (nutritional quality score)	1.8 (0.9)	1.6 (0.8)	1.9 (0.9)	1.5 (0.9)	2.1 (0.7)	1.9 (0.8)	<.01^b^	.57
Potatoes (nutritional quality score)	2.4 (1.5)	1.6 (1.6)	2.7 (1.7)	1.5 (1.5)	2.2 (1.3)	1.9 (1.1)	.71	.97
Meat (g/week)	2367.9 (1737.8)	2428.8 (1809.2)	2422.8 (1336.4)	2473.0 (1605.4)	2584.9 (1650.3)	2280.3 (1300.9)	.65	.50
Fish (g/week)	348.8 (701.5)	337.5 (710.7)	223.1 (242.1)	229.4 (267.8)	218.9 (232.9)	257.4 (237.1)	.61	.64
Eggs (g/week)	209.5 (305.1)	211.9 (265.7)	263.8 (210.6)	248.8 (250.0)	233.8 (163.6)	212.2 (200.1)	.40	.76
Dairy (g/day)	314.6 (573.5)	327.0 (800.3)	308.3 (301.5)	284.0 (383.7)	197.3 (193.3)	159.1 (172.0)	.49	.68
Nuts (g/day)	15.1 (22.7)	16.2 (19.9)	12.9 (16.4)	15.3 (18.3)	8.3 (10.3)	11.7 (13.1)	.11	.80
Spreadable fats (nutritional quality score)	1.1 (0.9)	1.1 (0.8)	1.2 (1.0)	1.1 (0.8)	1.1 (0.8)	1.0 (0.8)	.65	.91
Cooking fats (nutritional quality score)	1.5 (0.6)	1.2 (0.7)	1.3 (0.7)	1.3 (0.8)	1.4 (0.8)	1.3 (0.8)	.32	.41
Sweet snacks (g/week)	77.6 (83.3)	64.5 (89.6)	83.4 (83.9)	65.4 (56.4)	94.3 (68.6)	62.2 (60.6)	<.01^b^	.59
Savory snacks (g/week)	55.6 (74.0)	48.0 (67.6)	41.8 (37.4)	35.0 (37.0)	54.3 (49.4)	36.5 (47.8)	<.01^b^	.47
Ready-made meals (g/week)	127.3 (218.5)	77.4 (114.7)	112.9 (85.9)	78.2 (71.2)	98.8 (97.2)	80.0 (88.3)	.01^b^	.66
Sauces and gravy (g/week)	15.0 (20.6)	6.7 (9.1)	24.5 (22.8)	13.8 (18.2)	17.0 (17.1)	9.9 (14.3)	<.01^b^	.70
Alcohol (U/day)	0.7 (1.1)	0.7 (1.2)	0.7 (1.2)	0.5 (0.9)	0.5 (0.8)	0.5 (0.7)	.66	.66
Soft drinks and fruit juice (g/day)	209.4 (374.2)	212.1 (345.9)	176.1 (272.0)	204.8 (341.9)	278.3 (489.4)	218.3 (329.9)	.77	.66
Tea (g/day)	453.1 (448.1)	536.6 (504.6)	360.7 (390.7)	403.7 (433.0)	400.2 (399.0)	467.2 (464.2)	.03^b^	.95
Coffee (g/day)	424.7 (476.0)	396.3 (449.2)	338.8 (378.1)	303.6 (384.7)	403.5 (451.5)	280.9 (277.5)	.02^b^	.20

^a^Nutritional quality scores reflect factors such as whether whole wheat versus multigrain products were consumed or whether olive oil versus butter is used for frying. A lower score represents a healthier food choice. Extensive descriptions of these nutritional quality scores are attached as Multimedia Appendix 4.

^b^Significant values (ie, <.05).

### Sex Differences and Correlations in Body Composition Changes

**Figure 6:**
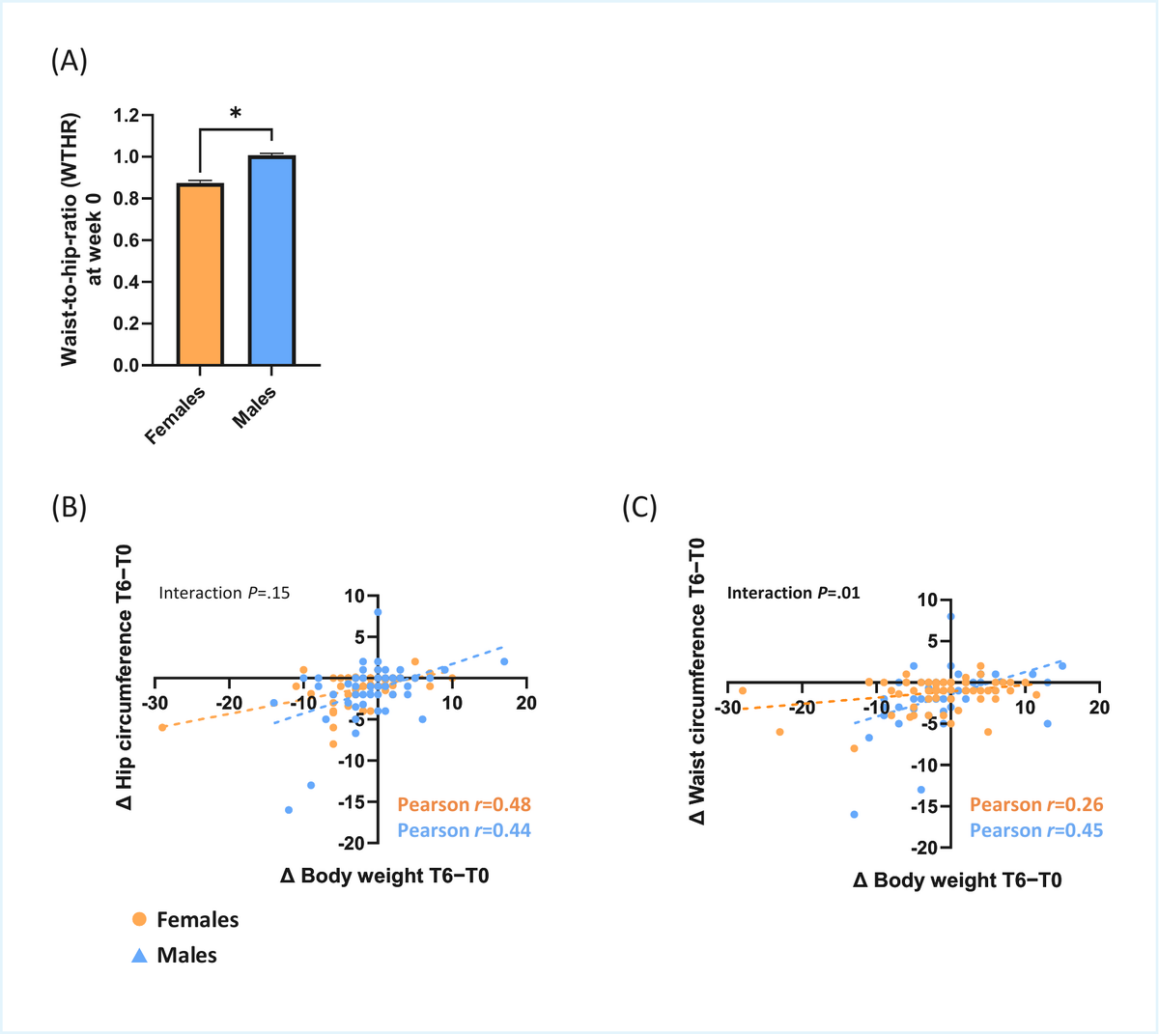
Sex differences in anthropometric measurements and fat mass loss. (A) Sex differences in waist-to-hip ratio at week 0, assessed fully online; data are expressed as means (SDs). Sex-specific correlations between Δ body weight and Δ waist circumference (B) and Δ hip circumference (C). In all correlation analyses, Δ values were calculated as the difference between measurements at the start and end of the 6-week trial. *P<.05.

Exploratory analyses revealed that the waist-to-hip ratio was higher in males compared with females (Figure 6A). Furthermore, weight loss correlated similarly with loss in hip circumference in females (*r*=0.48, *P*<.001) and males (*r*=0.44, *P*<.001), as no significant interaction with biological sex was detected (*P*=.15; Figure 6B). Body weight loss correlated differently with loss in waist circumference in females (*r*=0.26, *P*=.02) and males (*r*=0.44, *P*<.001), as a significant interaction with biological sex was detected (*P*=.01; Figure 6C).

## Discussion

### Principal Findings

The primary aim of this study was to evaluate the feasibility of conducting a fully remote, fully digital nutritional intervention in adults with overweight or obesity. Our findings demonstrate that such a digital-first approach is feasible: most participants reported that anthropometric measurements were easy to perform at home, the digital questionnaires were clear and understandable, and the reliability of self-reported measurements was supported by consistent correlations among changes in body weight, waist circumference, and hip circumference (Figure 3). Together, these results indicate that participants were able to self-collect anthropometric data of sufficient quality for use in a randomized controlled trial.

The secondary aim of the study was to assess whether meaningful anthropometric changes could be detected using self-collected data. Participants who received personalized nutritional advice lost more body weight than those receiving generic advice (Figure 5), confirming the greater effectiveness of personalized advice even in a fully digital format. This effect was not explained by differences in the ease of implementing the advice, which increased only when personalized food boxes were provided in addition to the advice (Figure 4). The addition of personalized food boxes further increased weight loss and reduced hip circumference more than either form of advice alone. Taken together, these results indicate that nutrition studies can be effectively conducted in a real-life, fully remote setting, achieving measurable intervention effects while maintaining ease of participation and high-quality data collection.

Exploratory analyses provided additional insights into sex differences in anthropometric responses. As expected from prior literature, males had higher waist-to-hip ratios and showed stronger correlations between weight loss and reductions in waist circumference compared with females. These findings support the biological plausibility of the self-measured data and highlight potential sex-specific patterns in responsiveness to nutritional interventions.

### Comparison With Prior Work

An important factor underlying the success of personalized nutrition is the tailoring of the information provided to the recipient [27], which makes the advice perceived as personally relevant, for which face-to-face contact could be an important contributor [16,17]. Digital delivery of advice lacks this potentially important component, which may reduce its effectiveness. Importantly, in this study, we show that although all advice was provided digitally without any face-to-face contact, personalized nutritional advice still led to a significant loss of body weight, whereas generic nutritional advice did not produce a statistically significant effect on body weight or other anthropometric measurements (Figure 5A). This finding is consistent with other studies comparing digitally provided generic versus personalized advice in populations with obesity [28-31]. Together, the findings of these studies and our study provide evidence for the added value of personalized nutritional advice compared with generic advice, even when delivered online. Yet, it remains a topic of discussion whether the same personalized nutritional advice would have been more effective if delivered in person, rather than online. Interestingly, one study compared the effect of delivering personalized nutritional advice online with or without a personal coach [32]. Participants receiving similar advice through an online coach demonstrated greater engagement with the program and had an increased likelihood of achieving clinically relevant weight loss. This finding suggests that receiving feedback from an actual human can enhance protocol adherence, possibly due to an increased sense of accountability, motivation, and perception of receiving care tailored to an individual’s specific needs. Similarly, another study found that offline nutritional advice was more effective than online advice in a small sample of rugby athletes [25], highlighting the potential added benefit of in-person delivery [33]. However, conventional face-to-face counseling also has its downsides, as it is more expensive, less flexible in terms of time and location, and ultimately less scalable compared with online methods [34]. Consequently, both online and in-person approaches have their own strengths, and one may be more appropriate than the other depending on an individual’s personal preferences and needs.

Interestingly, participants in the PP-group, who received personalized food boxes based on their personalized advice, were able to lose more body weight compared with the other two groups. This effect was not due to differences in physical activity, as PAL did not change in any of the groups (Table 3). This outcome may reflect the difficulty of translating personalized nutritional advice into an actual diet over a prolonged period. Indeed, these participants reported greater ease in following the personalized nutritional advice, as well as a greater perceived dietary change, compared with participants in the control and P-groups (Figure 4A and 4C). This novel finding suggests that future interventions may benefit from placing particular emphasis on helping participants translate their advice into practice, for example, by simplifying meal planning and reducing decision fatigue. These data also revealed that participants in the P-group did not find it easier or more enjoyable to implement their personalized advice compared with participants in the control group receiving generic advice (Figure 4A and 4B). However, participants in the P-group did achieve a significant loss of body weight, which was not observed in the control group (Figure 5A). These findings suggest that the success of personalized nutritional advice is not necessarily due to greater ease or enjoyment of implementation, which would be expected to increase protocol adherence. Instead, a better fit and higher quality of the advice are likely the main contributors to the enhanced effectiveness of personalized nutritional advice.

Habitual food intake was also measured, which indicated that all intervention groups changed their dietary habits in a similar way. All groups reduced the intake of ready-made meals (113.6 g vs 78.5 g, –30.9%); sauces and gravy (18.8 g vs 10.0 g, –46.8%); sweet snacks (84.8 g vs 64.1 g, –24.4%); savory snacks (50.5 g vs 40.0 g, –20.1%); bread, pasta, rice, and wraps (nutritional quality score of 1.9 vs 1.7, –10.5%); and vegetables (129.0 g vs 118.7 g, –8.0%); and replaced coffee with tea (Table 4). These results indicate that the intake of unhealthy food groups was particularly reduced, while the intake of healthy food groups such as fruit, legumes, nuts, and fish was maintained. Only vegetable intake decreased, but to a lesser extent (129.0 g vs 118.7 g, –8.0%). Fiber intake was also maintained and did not change (Multimedia Appendix 5). This suggests that all 3 intervention groups shifted their dietary intake toward a healthier habitual pattern, in line with the nutritional advice provided. No significant time × group interaction effects were found in the habitual food intake data. This is noteworthy, as the diet of the PP-group changed substantially—they received personalized food boxes for all meals throughout the study—while the control group and P-group did not receive such food boxes. Based on this approach, we hypothesized that the PP-group would show greater changes in habitual food intake compared with the other groups; however, these changes were not detected. This suggests that participants in the PP-group may have had difficulty accurately recalling the foods they consumed. In contrast to the habitual food intake data, the anthropometric data revealed group-specific effects of the nutritional interventions. For these reasons, we suspect that the collected habitual food intake data were not accurate enough to detect group-specific effects. Therefore, future studies may benefit from incorporating new technology-based dietary assessment tools that combine web-based programs with mobile apps and wearable devices [35]. Interestingly, legume intake showed a divergent pattern between groups, with a decrease in the control group and an increase in both intervention groups. Although this difference did not reach conventional statistical significance (interaction *P*=.051), the trend suggests a potential effect of the personalized interventions. Participants in the P- and PP-groups received targeted advice regarding protein and macronutrient intake, which may have encouraged higher legume consumption, whereas the control group received only general dietary guidance. Moreover, although the between-person variability in habitual food intake was considerable, this is not unexpected in nutritional intervention studies. Such variability likely reflects differences in individual dietary habits, adherence, and reporting accuracy. This observation emphasizes the importance of personalized dietary assessment and highlights the inherent heterogeneity in dietary behavior, even within relatively homogeneous study populations.

Sex differences in fat tissue distribution and fat loss across the waist and hip regions were observed in this fully remote study. Consistent with the literature, females had a lower waist-to-hip ratio compared with males (Figure 6A) [36]. Notably, body weight loss correlated more strongly with waist circumference loss in males (*r*=0.45) than in females (*r*=0.26, interaction *P*=.01; Figure 6B), which is also consistent with previous findings [37]. This sexual dimorphism is likely explained by the fact that males store more fat in the waist region compared with females, making the waist region more responsive to weight loss [37]. Together, these findings provide evidence for the accuracy of remotely collected data and demonstrate its potential to study sex differences in responses to nutritional interventions.

### Strengths and Limitations

Strengths of the study included its 3-arm design, with a total of 122 participants who were randomized and completed the collection of real-world study data, providing an ecological sample. This design allowed for the evaluation of the effectiveness of the nutritional interventions, albeit in a setting that was less controlled compared with a classical nutritional intervention study. However, there were some limitations associated with this study. First, no comparison was made with nondigital nutritional interventions. Such a comparison would have been valuable, as it could reveal potential differences in the direct effect size of online versus offline personalized nutrition advice. Future studies could include a nondigital control group to address this. Second, the study duration was 6 weeks, reflecting only short-term effects. The long-term impact of the interventions remains unknown, and future research with longer follow-up is needed to assess sustained effects. Third, due to dropouts, the PP-group included a relatively higher proportion of males (Table 1). This likely resulted in a slightly higher baseline body weight in the PP-group compared with the other groups, although the difference was not statistically significant. Randomization and statistical adjustments were applied to mitigate this imbalance, but future studies could aim for larger sample sizes to avoid similar discrepancies. Fourth, although this study demonstrated short-term beneficial effects of personalized nutritional interventions, these results do not guarantee long-term benefits, such as sustained weight loss. Previous studies have shown that maintaining weight loss is challenging, with many individuals regaining weight within 2-5 years [38,39]. In addition, long-term success is influenced not only by individual behavior change but also by broader factors, such as the food environment and supportive public health policies [40], which were beyond the scope of this study. Future research should therefore investigate whether personalized digital interventions can contribute to sustainable behavior change when combined with strategies addressing environmental and policy-level determinants. Fifth, a potential bias in studies relying on self-reported outcomes is participants’ tendency to report behaviors that align with perceived study goals, such as weight loss. To minimize this potential bias, we provided standardized instructions for anthropometric measurements, used validated dietary and activity questionnaires, and emphasized the importance of accurate reporting. The randomized design helped distribute any residual bias across study arms. In the food-provision arm, reporting bias may have been slightly higher due to participants’ awareness of the intervention; however, randomization and the use of multiple anthropometric measures (body weight, waist, and hip circumference) enhanced the robustness of the results. Cross-validation analyses (Figure 3) and sex-specific effects (Figure 6), consistent with prior literature, further support the reliability of our findings. Sixth, a sample size of 150 at the start of the intervention was considered sufficient to demonstrate the effectiveness of the personalized nutritional interventions. However, due to a relatively high number of dropouts (n=43 due to noncompliance and an additional n=15 due to outlier exclusion), only 122 participants were included in the final data analysis. This reduction in sample size may have limited the statistical power to detect the intended effects. While a dropout rate of 10%-20% was anticipated, the actual dropout due to noncompliance was 23.9% (43/180 participants), with an additional 8.3% (15/180 participants) excluded as outliers. This relatively high attrition rate may be attributed to the remote digital study design without any face-to-face contact and may also reflect a relatively high burden of participation. Data from noncompliant participants were excluded from the analysis, and it is important to acknowledge that the exclusion of participants who did not complete the procedures may have led to an overestimation of feasibility outcomes. Moreover, the high number of outliers could reflect increased misreporting, possibly resulting from unclear instructions. In future studies, real-time plausibility checks could be helpful to flag implausible values and prompt participants to reenter their measurements. Additionally, digital-first studies should account for potentially higher dropout rates and provide clear participant instructions. Despite the attrition, the final sample remained balanced across the 3 study arms, with 39-43 participants per group, allowing for meaningful comparisons of intervention effects. Seventh, because 91 individuals were excluded before participation, the feasibility results reflect only those who entered and completed the study. This selective inclusion may limit generalizability and could lead to an overestimation of feasibility in the broader target population.

### Future Directions

While personalized nutrition approaches hold promise in tailoring dietary advice to individual characteristics and improving adherence, it is important to recognize that individual choices are embedded within a broader socioecological context. Dietary behavior is shaped not only by biological and psychological factors but also by the surrounding food environment, which includes the availability, affordability, accessibility, and cultural norms of food consumption. A growing body of public health literature emphasizes the importance of considering food systems and food environments when designing interventions, as these structural factors often constrain or facilitate the adoption of individualized recommendations [12]. Furthermore, diets are a key driver of environmental change, linking nutrition with planetary health challenges, such as climate change, biodiversity loss, and unsustainable land and water use [41]. Integrating personalized approaches with strategies that improve local food environments and align with sustainable food system goals may therefore offer a more comprehensive and equitable pathway for improving dietary behavior and long-term health outcomes. Moreover, factors such as socioeconomic status, language barriers, and digital literacy can influence both access to and adherence with personalized digital nutrition advice. For example, populations living in socioeconomically deprived neighborhoods often face cumulative disadvantages, including unhealthy food environments, financial constraints, and limited digital access, which can undermine the effectiveness of digital health tools [42]. These considerations suggest that personalized nutrition approaches are likely to be most effective when coupled with efforts to reduce structural barriers and enhance inclusivity, such as tailoring content to different languages, improving accessibility for individuals with lower digital skills, and embedding interventions within supportive community and policy contexts.

### Conclusions

We conclude that it is feasible to conduct a fully remote, fully digital nutritional intervention study. Participants were able to independently perform anthropometric measurements at home, reported positive user experiences, and generated self-collected data of sufficient internal validity to detect meaningful changes over time. In addition, the study demonstrated that personalized nutritional advice led to greater weight loss than generic advice, even in the absence of face-to-face contact. The provision of personalized food boxes further facilitated the translation of advice into daily dietary behavior, resulting in the largest reductions in body weight and hip circumference. Taken together, our findings indicate that fully online nutritional intervention studies can be successfully implemented and offer a scalable approach for reaching broader populations while still producing reliable and actionable outcomes.

## Appendix

Multimedia Appendix 1 Questionnaires used in this study to (1) screen participants for inclusion and exclusion criteria, (2) collect baseline data, and (3) obtain feedback on the online intervention.

Multimedia Appendix 2 Examples of participant feedback, along with the generic and personalized nutritional advice provided.

Multimedia Appendix 3 CONSORT-eHEALTH checklist (V 1.6.1).

Multimedia Appendix 4 Overview of the calculation of nutritional quality scores.

Multimedia Appendix 5 Overall fiber intake during the study.

## Abbreviations

CONSORT: Consolidated Standards of Reporting Trials
FAO: Food and Agriculture Organization
PAL: physical activity level
P-group: personalized intervention group
PP-group: personalized plus intervention group
TNO: Netherlands Organization for Applied Scientific Research
UNU: United Nations University
WHO: World Health Organization

## Data Availability

Data are available from the corresponding author (SW) upon reasonable request.
