# Supplementary material for: A Real-Life Digital Intervention for Personalized Nutrition in Adults With Overweight or Obesity: Remote Randomized Controlled Trial
Source: J Med Internet Res. 2026 Jan 5;28:e73367. doi: 10.2196/73367 (PMC12817035; doi:10.2196/73367)
Supplement: Multimedia Appendix 2 [file jmir_v28i1e73367_app2.docx]

**Personal feedback**

**All participants received personal feedback based on anthropometric (A) do-it-yourself measurements, fiber related questions (Q) or physical activity level (PAL) related questions (for an overview see Supplemental file 1). Based on pre-defined cut-off values for categories participants received feedback as described in the table below. The amount of daily kcal was calculated based on basal metabolic rate (BMR) * PAL factor. BMR + 11.936 * weight (kg) + 587.728 * (length / 100)-8.129*age + 191.027*(Male=1; Female=0) + 29.279.**

| **Domain** | **Categories** | **Feedback** |
| --- | --- | --- |
| **Physical activity level (PAL) (Q)** | **Inactive  (PAL factor 1)** | **You hardly move at all. Do you know that sedentary behavior has negative health effects? Every form of physical activity counts and has a positive effect.** |
|  | **Sedentary  (PAL factor 2)** | **You do light physical activity. Do you know that every form of physical activity counts and has a positive health effect. So exercising is good, but increasing your physical activity pattern is even better.** |
|  | **Moderately active (PAL factor 3)** | **You are doing moderate physical activity. Super. This means that you exercise enough. Do you know that exercising is very healthy and that the positive health effects of exercise work along in a continuum. This means that more exercise is always better, whether more often, longer or more intensively.** |
|  | **Vigorously active (PAL factor 4)** | **You do heavy physical activity. Super. This means that you exercise a lot and intensively and that is very healthy. The positive effects of exercise work along in a continuum. This means that more exercise is always better, whether more often, longer or more intensively. Of course it is important to maintain your heavy physical activities in combination with good nutrition.** |
|  | **Extremely active  (PAL factor 5)** | **You do strenuous physical activity. Wow! This means that you exercise a lot and very intensively which is very healthy. Of course, it is very important that you maintain your strenuous physical activity with good nutrition so that you do not exhaust your body and it can recover properly.** |
| **Fiber intake (Q)** | **Low** | **Your fiber intake is lower than the recommended amount of fiber. Vegetables, fruit, potatoes, whole wheat bread, breakfast cereals, legumes and nuts contain a lot of dietary fiber.** |
|  | **Normal** | **Your fiber intake is according to the recommendations, keep this up. Vegetables, fruit, potatoes, whole wheat bread, breakfast cereals, legumes and nuts contain a lot of dietary fiber.** |
|  | **High** | **Your fiber intake is very good, keep this up. Vegetables, fruit, potatoes, whole wheat bread, breakfast cereals, legumes and nuts contain a lot of dietary fiber.** |
| **BMI (A)** | **Normal** | **Your weight is healthy. Nice. Continue to eat healthy and exercise enough to keep it that way.** |
|  | **High** | **Your weight is too high. Try to lose weight responsibly or at least not gain weight by eating healthy and exercising sufficiently. Your waist circumference is also important for optimal advice. Therefore, also measure your waist circumference.** |
|  | **Very high** | **Your weight is much too high. It is better for your health to lose weight responsibly or at least not to gain weight by eating healthy and exercising sufficiently. Your waist circumference is also important for optimal advice. Therefore, also measure your waist circumference.** |
| **Waist circumference (A)** | **Low** | **Your waist circumference is low. This puts you at an increased risk of malnutrition. Try to increase weight waist responsibly under supervision.** |
|  | **Normal** | **Your waist circumference is healthy. Try to keep your waist circumference that way** |
|  | **Increased** | **Your waist circumference is increased. Keep an eye on your waist circumference and check your BMI and body shape.** |
|  | **High** | **Your waist circumference is too high. Try to lose weight responsibly under supervision.** |
| **Body shape (A)** | **Apple** | **In people with an apple body shape, fat storage is mainly located on the abdomen and in the organs. This puts you at greater risk of developing cardiovascular disease especially in combination with a high BMI and/or waist circumference.** |
|  | **Pear** | **Pear-shaped body shape typically have a flat stomach. The fat storage is mainly located around the hips and buttocks. This is beneficial because it prevents fat from being stored in the organs. Pear-shaped body types are therefore less likely to develop diabetes and cardiovascular disease.** |
|  | **OK** | **Your height to waist ratio is in the right proportion. This means that you have a healthy amount of body fat nicely distributed over your body. This lowers your risk of cardiovascular disease.** |
|  | **Carrot** | **Carrot body shapes are slim or have little body fat or muscle mass and are more at risk of being underweight and/or losing muscle mass or muscle function. It is important to continue to maintain the function of your muscles with your diet.** |
| **Body type (A)** | **Ectomorph** | **You have an ectomorph body type. An ectomorph body type is often described as a body type that is slim, with long limbs and a low body fat percentage. People with an ectomorphic body type often find it difficult to gain weight or increase muscle mass. This is because people with an ectomorph body type generally have a fast metabolism.** |
|  | **Mesomorph** | **You have a mesomorph body type. A mesomorph body type is often described as a muscular and athletic. People with this body type typically have broad shoulders, a narrow waist, and well-defined musculature. People with a mesomorph body type often find it relatively easy to increase and maintain muscle mass and keep fat mass under control.** |
|  | **Endomorph** | **You have an endomorph body type. An endomorph body type is often described as a sturdy build. Individuals with an endomorphic body type generally have a broader build with wider hips (curvy) and generally have a relatively short height. The body fat percentage is often higher than average and can easily increase. Usually, individuals with an endomorph body type are strong.** |
| **kcal** |  | **Your daily energy requirement is … kcal** |

**Advice for the control group**

In this advisory, the Health Council outlines which foods and dietary patterns contribute to health benefits. The council systematically evaluated scientific knowledge on the relationship between diet and chronic diseases. Based on this, the new Guidelines for a Healthy Diet were formulated:

- Follow a dietary pattern that is more plant-based and less animal-based, in line with the guidelines below.
- Consume at least 200 grams of vegetables and at least 200 grams of fruit daily.
- Eat at least 90 grams of whole-grain bread, whole-grain products, or brown bread daily.
- Include legumes in your diet weekly.
- Consume at least 15 grams of unsalted nuts per day.
- Have a few servings of dairy products daily, such as milk or yogurt.
- Eat fish once a week, preferably fatty fish.
- Drink three cups of tea daily.
- Replace refined grain products with whole-grain products.
- Substitute butter, hard margarine, and solid cooking fats with soft margarine, liquid cooking fats, and vegetable oils.
- Replace unfiltered coffee with filtered coffee.
- Limit the consumption of red meat, particularly processed meat.
- Drink as few sugary beverages as possible.
- Avoid alcohol or limit consumption to no more than one glass per day.
- Limit salt intake to a maximum of 6 grams per day.
- Nutritional supplements are not necessary, except for specific groups for whom a supplementation recommendation applies.

**Example advice for the personalized intervention group**

**Dear (Customer Name),**

The first steps toward your ideal diet have been made!

**Dietary Plan**
Based on the test results, your daily energy requirement is 2162 kcal.
This means your ideal daily menu composition consists of:

- **Carbohydrates:** 50%
- **Proteins:** 20%
- **Fats:** 30%

Additionally, it’s important to drink at least 1.5 liters of water per day. Avoid soft drinks and juices. You can also drink coffee and/or tea without sugar.
To create your daily menu based on this macronutrient ratio and energy requirement, you can use the following tool: <https://www.voedingscentrum.nl/nl/thema/apps-en-tools-voedingscentrum/mijn-eetmeter-app-online.aspx>

Since your personal goal is to lose weight, we recommend consuming 400 kcal less than your energy requirement, which means eating a maximum of 1762 kcal per day. This might result in a slight feeling of hunger. If this happens, try drinking a glass of water first and engaging in a distracting activity. It also helps to take your time with meals or snacks, as it takes about 20 minutes for the feeling of fullness to kick in. Hopefully, this will make it easier for you to stay on track!

**What are Macronutrients?**

**Carbohydrates** provide your body with energy. It’s best to consume them in forms that are proven to offer health benefits, such as whole-grain products, potatoes, legumes, vegetables, and fruits.

**Proteins** are both a nutrient and a building block for your body. They can be of animal origin (meat, fish, milk, cheese, and eggs) or plant-based (whole-grain bread, legumes, and nuts). Proteins also promote a feeling of fullness, making it easier to lose weight by reducing hunger.

**Fats** are a source of energy, various vitamins such as A, D, and E, and essential fatty acids that are crucial as building blocks for your body.

Can’t wait to get started on your healthy lifestyle? **Start today!**

**Example advice personalized intervention plus group**

**Dear (Customer Name),**

The first steps toward your ideal diet have been taken!

**Dietary Plan**
Based on the test results, your daily energy requirement is 2162 kcal. This means your ideal daily menu composition consists of:

- **Carbohydrates:** 50%
- **Proteins:** 20%
- **Fats:** 30%

Additionally, it’s important to drink at least 1.5 liters of water per day. Avoid soft drinks and juices. You can also drink coffee and/or tea without sugar.

**The meal box you receive is tailored to this advice.**

You will receive information about the delivery of your meal box via email. Try to spread the meals evenly across the eating moments of the day, for example:

- **7:00** Breakfast
- **10:00** Snack
- **12:30** Lunch
- **15:00** Snack
- **18:00** Dinner
- **20:00** Evening snack

On the days when you don’t receive a box, you can create your own daily menu based on the macronutrient ratio and your energy requirements using this app:

<https://www.voedingscentrum.nl/nl/thema/apps-en-tools-voedingscentrum/mijn-eetmeter-app-online.aspx>.

Since your personal goal is to lose weight, we recommend consuming 400 kcal less than your energy requirement, which means eating a maximum of 1762 kcal per day. This might result in a slight feeling of hunger. If this happens, try drinking a glass of water first and engaging in a distracting activity. It also helps to take your time with meals or snacks, as it takes about 20 minutes for the feeling of fullness to kick in. Hopefully, this will make it easier for you to stick with it!

**What are Macronutrients?**

**Carbohydrates** provide your body with energy. It’s best to consume them in forms that are proven to offer health benefits, such as whole-grain products, potatoes, legumes, vegetables, and fruits.

**Proteins** are both a nutrient and a building block for your body. They can be of animal origin (meat, fish, milk, cheese, and eggs) or plant-based (whole-grain bread, legumes, and nuts). Proteins also promote a feeling of fullness, making it easier to lose weight by reducing hunger.

**Fats** are a source of energy, various vitamins such as A, D, and E, and essential fatty acids that are crucial as building blocks for your body.

**Can’t wait to start your healthy lifestyle?** Start today!
