# Supplementary material for: A Real-Life Digital Intervention for Personalized Nutrition in Adults With Overweight or Obesity: Remote Randomized Controlled Trial
Source: J Med Internet Res. 2026 Jan 5;28:e73367. doi: 10.2196/73367 (PMC12817035; doi:10.2196/73367)
Supplement: Multimedia Appendix 4 [file jmir_v28i1e73367_app4.docx]

#### ****Nutritional quality score is categorized based on color**** **Red** **= This color is the worst category, indicating unhealthy food choices.**

#### **Orange = This color is the medium category, indicating there is room for improvement.** **Green** **= This color is the best category, indicating the healthiest food choice.**

#### **Grey** **= This is a neutral category, indicating that the respective food is not being consumed or used.**

#### ****Bread****

0 – Grey – Not eaten
1 – Green – Only whole wheat
2 – Orange – Both whole wheat and white/brown/multigrain
3 – Red – Only white/brown/multigrain

#### ****Pasta/Rice/Wraps****

0 – Grey – Not eaten
1 – Green – Only whole wheat pasta, brown rice, whole wheat wraps
2 – Orange – Both whole wheat and white/multigrain
3 – Red – Only white/multigrain pasta, rice, wraps

#### ****Potatoes****

0 times per week – Orange
1-2 times per week – Green
3-4 times per week – Orange
5-7 times per week – Red

#### ****Spreadable fats****

0 – Grey – Not used
1 – Green – Only soft butter (e.g. low-fat margarine, halvarine)
2 – Orange – Both soft and hard butter
3 – Red – Only hard butter (e.g. regular butter)

#### ****Cooking fats****

0 – Grey – Not used
1 – Green – Only liquid cooking oil (e.g. sunflower oil, olive oil)
2 – Orange – Both liquid oil and solid cooking fat
3 – Red – Only solid cooking fat (e.g. regular butter)
