# Supplementary material for: A Real-Life Digital Intervention for Personalized Nutrition in Adults With Overweight or Obesity: Remote Randomized Controlled Trial
Source: J Med Internet Res. 2026 Jan 5;28:e73367. doi: 10.2196/73367 (PMC12817035; doi:10.2196/73367)
Supplement: Multimedia Appendix 5 [file jmir_v28i1e73367_app5.docx]

## Supplementary table: Overall fiber intake during the study.

| **Fiber intake (g)** | **Week 0** | **Week 2** | **Week 4** | **Week 6** |
| --- | --- | --- | --- | --- |
| Control group | 11.3 ± 0.8 | 12.2 ± 1.2 | 13.0 ± 1.0 | 11.6 ± 0.9 |
| Personalized intervention (P-group) | 12.4 ± 1.3 | 14.8 ± 1.9 | 13.7 ± 1.6 | 12.5 ± 1.2 |
| Personalized Plus intervention (PP-group) | 12.5 ± 1.7 | 11.4 ± 1.3 | 11.1 ± 1.0 | 12.1 ± 1.5 |
